# Bilateral Simultaneous Tibial Tubercle Avulsion in an Adolescent Football Player with Previous Bilateral Osgood–Schlatter Disease

**DOI:** 10.1155/2019/8535370

**Published:** 2019-03-24

**Authors:** Jaime Dalla Rosa Nogales, José Juan Nogales Zafra

**Affiliations:** ^1^Complejo Hospitalario Integral Privado, Avda Carlos Haya 121, CP 29010, Spain; ^2^Chief of Orthopaedic Surgery and Traumatology Department, Complejo Hospitalario Integral Privado, Avda Carlos Haya 121, CP 29010, Spain

## Abstract

Tibial tubercle avulsion fractures are a very uncommon injury, accounting between 0.4 and 2.7% of all epiphyseal injuries. Bilateral lesions are extremely rare with only 20 cases described in the literature. They occur more frequently in male adolescents and during sport activities that require jumping and sprinting, such as football or basketball. We report the case of a 13-year-old boy who sustained simultaneous bilateral tibial avulsion fractures on the background of a previous conservatively managed bilateral Osgood–Schlatter disease.

## 1. Introduction

Tibial tubercle avulsion fractures are a very uncommon injury, accounting between 0.4 and 2.7% of all epiphyseal injuries [1]; however, the prevalence is uncertain due to the variations in the identification of this injury pattern, since many authors include them within the proximal epiphysiolysis of the tibia and others as a fracture-avulsion, and even the well-localized lesions are sometimes interpreted as variants of a lesion of Osgood–Schlatter [2]. Bilateral lesions are extremely rare with only 20 cases described in the literature [3]. They occur more frequently in male adolescents and during sport activities that require jumping and sprinting, such as football or basketball [4]. The development of the proximal epiphysis occurs through the fusion of the epiphyseal plate in late adolescence. This epiphysis is formed from the primary tibial diaphyseal and proximal epiphyseal ossification centers. The plate fuses completely between the ages of 13 and 15 years in girls and between the ages of 15 and 19 years in boys. The physeal closure progresses from the posterior to the anterior part of the tibia.

Therefore, the anterior part of the proximal tibia remains being a weaker region than the posterior part of the proximal tibia. The columnar cells are structurally weak, and therefore, cannot withstand tensile forces exerted by the knee extensor mechanism, which explains the occurrence of avulsion fractures in adolescents. These fractures can be associated with some predisposing conditions and diseases, such as osteogenesis imperfecta [5] and Osgood–Schlatter disease [4]. It can occur in two situations: sudden knee flexion against a contracted quadriceps muscle and, as in the present case, excessive contraction of the quadriceps during extension of the knee, both of which are common during sport activities [6]. Activities such as jumping and sprinting that require landing can lead to knee locking in extension and thus create the necessary biomechanical conditions for this type of fracture to occur [3]. Several classification systems have been described to categorize these fractures, which can be confusing to the reader given the use of subcategories [4]. The Watson-Jones classification [7] used the fracture location relative to the ossification centers with types I, II, and III being distal to the ossification center, through the ossification centers, and extending proximally through the joint, respectively. Ogden et al. [8] subdivided this classification into A (noncomminuted fractures) and B (comminuted fractures). Later, Ryu and Debenham [9] added a type IV fracture, defined as a fracture of the tibial epiphysis with posterior extension. Most recently, McKoy and Stanitski [10] suggested the addition of a type V two-part fracture, which is a “Y”-shaped fracture pattern in the proximal region of the knee to complete the classification system. In the same year, Davidson and Letts [11] suggested a nonphyseal variation of the type V fracture in which periosteal lifting of the patellar tendon insertion occurs, and it is associated with small subchondral bone fragments.

The complications associated with this pattern fractures include compartment syndrome (caused by bleeding from the anterior recurrent artery), knee stiffness, genu recurvatum (because of a deficit in the growth of the anterior tibia), and a high or low patella. Growth deficiency, although extremely rare, has also been described. Nonunion, malunion, infection, and pain have been reported. All complications associated with TT avulsion fractures are very uncommon [3].

We report the case of a 13-year-old boy who sustained simultaneous bilateral tibial avulsion fractures on the background of a previous conservatively managed bilateral Osgood–Schlatter disease, treated with some occasional injection. We consider reporting this case for its uniqueness and as an educational review of this pathology.

## 2. Case Presentation

**Figure 1:**
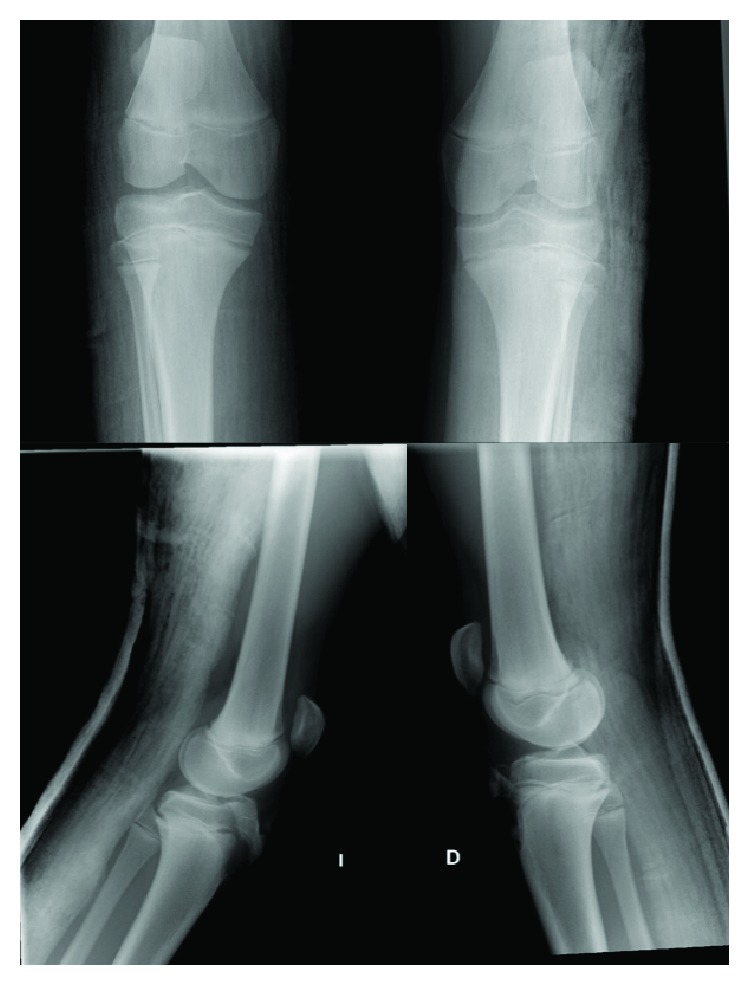
Plain radiographs of both knees. Right knee (D) showing an Ogden type IB fracture and the left one (I) type IIIA Ogden fracture.

**Figure 2:**
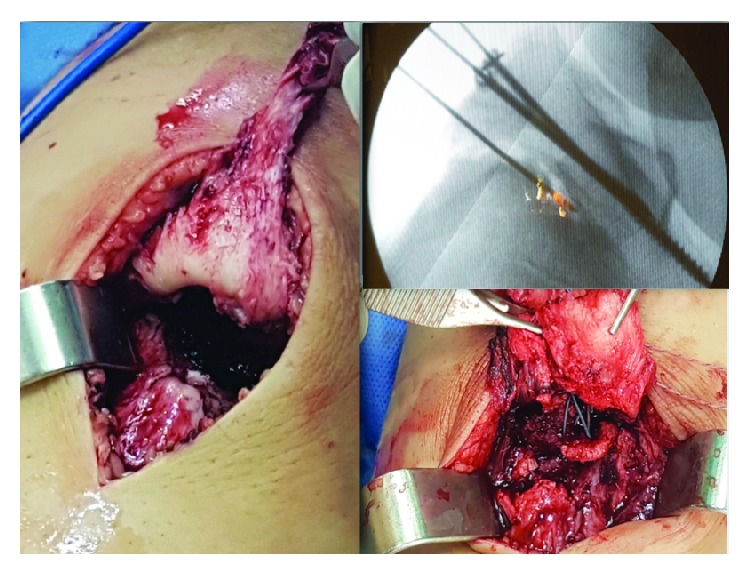
Intraoperative pictures showing approach and radiological control images.

**Figure 3:**
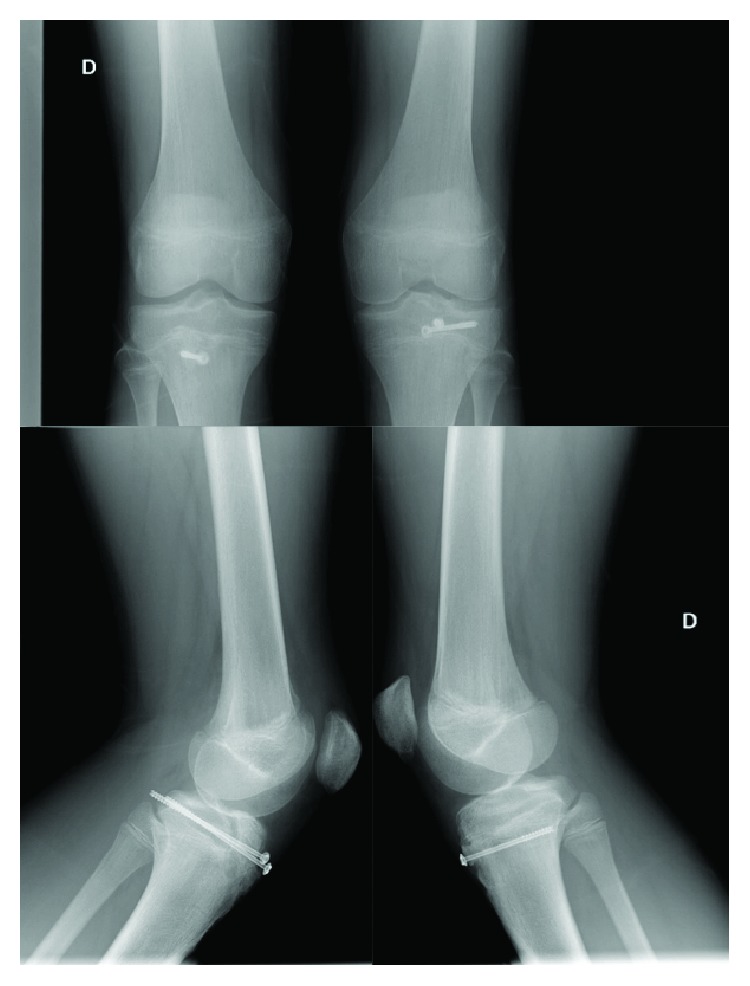
Plain radiographs of both knees at 6 weeks postoperative. Right knee (D) showing one 4.5 mm cannulated screw with a washer and the left one (I) two 4.5 mm cannulated screws with a washer.

A boy who was 13 years old presented to the emergency room with pain and difficulty in walking after a break (sudden deceleration) during a football match. The patient's height and weight were 1.62 m and 59 kg, respectively. Physical examination indicated pain when trying to fully extend either knees, bilateral pain on palpation, swelling over the anterior tibial tuberosity, joint effusion, and inability to walk. Ligament manoeuvres were negative (bilateral Lachman test). This patient has a previous history of Osgood–Schlatter disease in both knees. He suffered from anterior knee pain the year before. This pain forced him to stop sport activities and to undergo different treatments. He was treated with resting from contact-sport activities, physical therapy, and as a last resort, some injections (he was treated in a different hospital, and the patient cannot specify which drug was administered). Anteroposterior and lateral radiographs of both knees evidenced a bilateral tibial tuberosity avulsion fracture (Figure 1). The fractures of both knees were classified as Ogden type IIIA in the left knee and type IB in the right knee. Surgical treatment was proposed (open reduction and internal fixation with screws), and the patient underwent surgery 24 hours after the trauma. Under spinal anesthesia and in a supine position, an anterior approach was performed in both knees. Intravenous tranexamic acid was used in this case to avoid the use of bilateral tourniquet. After dissection and haematoma drainage, we cleaned the fracture site and reduced the distal fragment fracture under radiological control (Figure 2). The left knee (Ogden type IIIA) was internally fixed with 2 cannulated screws of 4.5 mm with a washer (Asniss III, Stryker, Selzach, Switzerland). The right one (type IB) was fixed with one cannulated screw and a washer. We reinforce fixation of both patellar tendons with one suture anchor (Iconix 2.3 mm, Stryker, Mahwah, NJ, USA) (Figure 3).

On the basis of the patient's weight, type of fracture, and bilateral occurrence, immobilization was indicated for 3 weeks using a bilateral knee brace. Physical therapy was started with passive range of motion (ROM) exercises, and after a 3-week-period, loading and quadriceps strengthening exercises were allowed. At 8 weeks, full loading and active ROM exercises were performed. At 12 weeks after surgery, the patient had already achieved full ROM (0°-140°) and regained bilateral knee extension strength.

**Figure 4:**
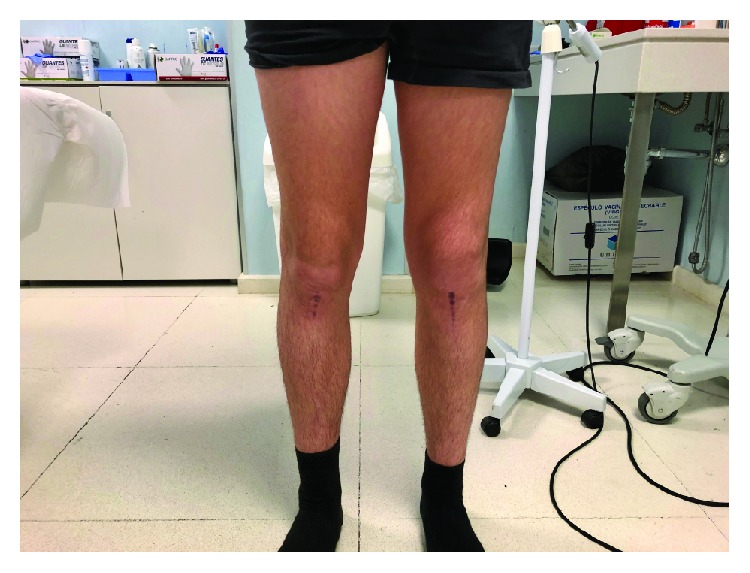
Patient at the office. Nonsymptomatic hypertrophic scar is appreciated.

Approximately 20 weeks after the surgery, the patient was able to perform physical activities without limitations at the same level as before the injury. Return to play was allowed at 6 months postoperative. A nonsymptomatic hypertrophic scar was developed, but the patient has no limitations during sport activities. By now, there has been no need for hardware removal (Figure 4).

## 3. Discussion

Bilateral tibial tubercle fractures are a very uncommon injury with only 20 cases reported in literature. In most cases, they occur during sport activities [12, 13]. The mechanism of injury and the type of lesion are age-dependent [14]. In childhood, the most frequent mechanism of injury is an abduction or adduction force (varus or valgus type); between 10 and 12 years of age, the most frequent mechanism of injury is a hyperextension trauma of the knee which occurs during jumping (hyperextension type), whereas in late adolescence, these fractures are extremely rare and are caused by a forced flexion of the knee during landing (flexion type).

Although the role of Osgood–Schlatter disease in the genesis of this injury is controversial [15], there are cases reported previously with this affection [2, 16–19]. Osteogenesis imperfecta also has been proposed as a predisponding factor. This case supports the hypothesis that patients with previous Osgood–Schlatter disease have a risk for developing tibial tuberosity avulsion and should be allowed for an adequate period of restricted activities, specially sport-related activities. Some authors [20] promote restricting sport activities until healing of diagnosed Osgood–Schlatter disease to prevent risk of the tibial tubercle fracture.

The decision on the type of treatment to be provided is made on the basis of deviation of the fracture. Fractures without deviation can be treated with splint immobilization for 4 weeks, followed by rehabilitation aimed at achieving a complete ROM within 8 weeks. Deviated and/or comminuted fractures require surgical treatment consisting of open reduction and internal fixation. Several types of surgical instruments for the treatment of these injuries have been reported in the literature, such as Kirschner wires, tension bands [20], suture anchors, cerclages, and cannulated screws. Treatment using cannulated screws is the most often cited and has the advantage of a faster rehabilitation. Closed reduction using percutaneous screws is another treatment option, indicated for nondisplaced and noncomminuted fractures [21]. Nicolini et al. [3] made a great review work with simultaneous bilateral injuries. They used three cancellous screws and placed them in an inverted triangular arrangement (two screws in the articular fragment and one in the tibial tubercle, all parallel to the physis). We chose this treatment method because it generates stability at the fracture site, allowing for a faster recovery.

All orthopaedic trauma surgeons should be aware of this avulsion fracture pattern. We must be aware of patients with Osgood–Schlatter disease and suggest to patients to restrict sport activities temporarily to prevent risk of fractures of the tibial tubercle. Once this occurs, an excellent outcome is achievable with surgical repair.

## References

[1] Ergün M., Taskiran E., Özgürbüz C. (2003). Simultaneous bilateral tibial tubercle avulsion fracture in a basketball player. *Knee Surgery, Sports Traumatology, Arthroscopy*.

[2] Mosier S. M., Stanitski C. L. (2004). Acute tibial tubercle avulsion fractures. *Journal of Pediatric Orthopaedics*.

[3] Nicolini A., Carvalho R., Ferretti M., Cohen M. (2018). Simultaneous bilateral tibial tubercle avulsion fracture in a male teenager: case report and literature review. *Journal of Pediatric Orthopaedics B*.

[4] Newman C., Musiienko D., Law S. (2017). Surgical fixation of bilateral simultaneous avulsion fractures of the proximal tibia in a 12-year-old with history of conservatively managed unilateral tibial avulsion fracture. *Case Reports in Orthopedics*.

[5] Khodadadyan-Klostermann C., Morren R., Raschke M., Haas N. (2003). Simultaneous bilateral tibial tubercle avulsion fractures in a boy with osteogenesis imperfecta: a case report and literature review. *European Journal of Trauma*.

[6] Roy S. P., Nag K. (2013). Simultaneous bilateral tibial tuberosity avulsion fractures in adolescence: case report and review of 60 years of literature. *Injury*.

[7] Watson-Jones R. (1955). *Fractures and joint injuries 4*.

[8] Ogden J. A., Tross R. B., Murphy M. J. (1980). Fractures of the tibial tuberosity in adolescents. *The Journal of Bone & Joint Surgery*.

[9] Ryu R. K. N., Debenham J. O. (1985). An Unusual Avulsion Fracture of the Proximal Tibial Epiphysis: Case Report and Proposed Addition to the Watson-Jones Classification. *Clinical Orthopaedics and Related Research*.

[10] McKoy B. E., Stanitski C. L. (2003). Acute tibial tubercle avulsion fractures. *Orthopedic Clinics of North America*.

[11] Davidson D., Letts M. (2002). Partial sleeve fractures of the tibia in children: an unusual fracture pattern. *Journal of Pediatric Orthopaedics*.

[12] Williams D., Kahane S., Chou D., Vemulapalli K. (2015). Bilateral proximal tibial sleeve fractures in a child: a case report. *Archives of Trauma Research*.

[13] Potenza V., Caterini R., Maglione P., Bisicchia S., Farsetti P. (2011). Simultaneous bilateral flexion-type Salter-Harris II fractures of the proximal tibia: a case report and review of the literature. *The Open Orthopaedics Journal*.

[14] Mubarak S. J., Kim J. R., Edmonds E. W., Pring M. E., Bastrom T. P. (2009). Classification of proximal tibial fractures in children. *Journal of Children's Orthopaedics*.

[15] Jalgaonkar A. A., Dachepalli S., Al-Wattar Z., Rao S., Kochhar T. (2011). Atypical tibial tuberosity fracture in an adolescent. *Orthopedics*.

[16] Takai S., Yoshino N., Kubo Y., Suzuki M., Hirasawa Y. (2000). Bilateral epiphyseal fractures of the proximal tibia within a six-month interval: a case report. *Journal of Orthopaedic Trauma*.

[17] Merloz P., de Cheveigne C., Butel J., Robb J. E. (1987). Bilateral Salter-Harris type II upper tibial epiphyseal fractures. *Journal of Pediatric Orthopaedics*.

[18] Chow S. P., Lam J. J., Leong J. C. (1990). Fracture of the tibial tubercle in the adolescent. *The Journal of Bone and Joint Surgery. British volume*.

[19] Levi J. H., Coleman C. R. (1976). Fracture of the tibial tubercle. *The American Journal of Sports Medicine*.

[20] Gowda N. B. S., Kumar M. J. (2012). Simultaneous bilateral tibial tubercle avulsion fracture in a case of pre-existing Osgood-Schlatter disease (OSD). *Journal of Orthopaedic Case Reports*.

[21] Ozkayin N., Aktuglu K. (2005). Avulsion fractures of tibial tuberosity in adolescents. Treatment with closed reduction and percutaneous screwing, using MRI to identify combined intraarticular lesions. *Saudi Medical Journal*.

